# Immunoinformatic-guided design of a polo-like kinase 1 (PLK1)-targeted multi-epitope vaccine with preliminary *in vitro* validation

**DOI:** 10.3389/fbinf.2026.1843200

**Published:** 2026-06-02

**Authors:** Gayatri Munieswaran, Nandha Kumar Subramani, Anand Prem Rajan, Venkatraman Manickam

**Affiliations:** School of Biosciences and Technology, Vellore Institute of Technology, Vellore, India

**Keywords:** cancer immunotherapy, immune receptors, immune simulation, multi-epitope vaccine, plk1

## Abstract

**Introduction:**

Polo-like kinase 1 (PLK1) is a well-established oncogenic protein, as indicated by elevated levels of protein expression and function in a variety of tumor malignancies, and thus helps in metastasis of the disease. To target the PLK1, the study designed a cancer antigen that is capable of activating enhanced immune signaling pathways using an immunoinformatic approach.

**Methodology:**

To achieve the study’s objective initially, the potential B- and T-cell epitopes were screened through IEDB resources. By analyzing immunological profiles, the highly antigenic, non-toxic and non-allergenic epitopes were screened and further utilized for vaccine design. Using appropriate linkers and adapters, the multi-epitope vaccine was constructed, and subsequent structural modelling and validation were analyzed. Following that, the immunogenic potential was analyzed through molecular docking, molecular dynamic simulations, and immune simulations. *In silico* cloning and preliminary *in vitro* validation using selected CTL epitopes were performed.

**Results:**

Four highly antigenic B-cell, five CTL, and five HTL epitopes along with linkers and 50s ribosomal adjuvants were utilized for vaccine construction. The subsequent physicochemical characterization and structural validation revealed that the PLK1 vaccine is considered highly stable. The molecular interactions with innate receptors revealed that the PLK1 vaccine had high binding affinity with the TLR4 receptor (−402.83 kcal/mol) compared to TLR2. The investigations under physiological conditions exhibited that the PLK1 vaccine with TLR complexes maintained structurally stable conformations. The elevated level of IFN-gamma and IL-2 productions was observed in the immune simulation analysis. The final recombinant length of the optimized gene expression was observed to be 6,488 bps. A decrease in PLK1 transcript levels was observed in the SKBR3 cells after treatment with specific CTL epitopes.

**Discussion:**

The enhanced immune profiles of the constructed PLK1 demonstrated a favourable predicted interaction with TLR4, suggesting a potential receptor recognition that may associated with Th1-mediated immune responses.

**Conclusion:**

Overall, the PLK1 cancer antigen may act as a promising candidate in the context of cancer immunotherapy. Further, experimental validation in proper animal models is required to validate its immunogenic potential.

## 1 Introduction

Cancer becomes the leading cause of mortality worldwide, and with resistance against treatment measures, it requires more efficient preventive and alternative therapeutic strategies. The World Health Organization (WHO) reported that approximately 20 million incidences and 9.7 million mortalities occurred in the year 2022. Among the cancer types, lung, breast and colorectal cancers remain in the top list of cancer incidences and deaths. Further, the study reported that global burden of cancers will be increased 77% with 35 million new cases in the next two decades (Bray et al., 2024; World Health Statistics, 2022). Due to its rapid evolution, complexity of diseases, and heterogeneity of the tumors, conventional methods such as chemotherapy, radiotherapy, surgical treatment and hormonal therapy often exhibited limited efficacy, adverse side effects and drug resistance (Shyam et al., 2023). This underscores the urgent need for targeted and personalized therapeutic approaches, such as developing vaccines in immunotherapy, which exploit the remarkable potential to recognize and eliminate tumor cells while evading the host immune system (Townsend et al., 2022; Braun et al., 2025). For the development of a vaccine, the tumor-associated antigens that are overexpressed in malignant tumors are represented as attractive targets. One such important oncogenic target that is escalating in various types of cancers is polo-like kinase 1 (PLK1).

PLK1 is recognized as a class of the serine/threonine kinase family that acts as a cruical protein in various cellular developmental processes that include spindle assembly, mitosis, cytokine signaling, centrosome maturation, apoptosis, and autophagy. The primary function of PLK1 is to activate the cyclic dependent kinase-1-cyclin B1 (CDK1-cyclin B1) complex in the cell cycle of G2/M transition (Munieswaran and Manickam, 2026). Despite the enormous role played by PLK1, the overexpression of this protein is found in a variety of tumors, which include skin, lung, breast, prostate, ovarian, and stomach cancers (Kahl et al., 2022; Peng et al., 2024; Guerrero-Zotano et al., 2023; Li et al., 2026; Reda et al., 2022). Precisely, the overexpression of the protein decreases the production of phosphate and Tensin homolog (PTEN). Thus, it leads to the activation of important oncogenic pathways such as phosphoinositide 3-kinase/protein kinase B/mammalian target of rapamycin (PI3K/AKT/mTOR) pathway as well as aberrantly activates the extracellular signal-regulated kinase pathway. The study reported that the dysregulation of PLK1 significantly inhibits the activity of tumor suppressor proteins like RE1-silencing transcription factor (REST) and tumor antigen (p53) through phosphorylation and driven tumorigenesis (Glaviano et al., 2023; Lashen et al., 2023). A previous study conducted on the oncogenic missense variants in human PLK1 established that PLK1 is frequently associated with poor prognosis and higher tumor grades, as well as decreases the overall survival outcome of cancer patients (Munieswaran and Manickam, 2025). Due to this, PLK1 is considered a promising oncogenic target that requires precise and effective therapeutic approaches in anticancer treatments.

The conventional methods that target PLK1, like the identification of small molecule inhibitors, often demonstrate anticancer activity, but their clinical utility is heavily limited by off-target effects, accumulation of systemic toxicity, and the development of drug resistances (Katta et al., 2023; Kim et al., 2022). Further, the absence of any commercial drug that targets PLK1 also highlights the challenges associated with the translational changes from preclinical to clinical applications. In pursuit of that, epitope-based peptide vaccine constructions offer a potential alternative strategy through eliciting targeted cellular and humoral immune responses against tumor-associated antigens (Kamali et al., 2025; Zhang et al., 2025; Prawiningrum et al., 2022). Notably, the advancements in immunoinformatic and computational biology further accelerated the rational designing of vaccines. Subsequently, the approach enables the identification of immunodominant T and B cell epitopes, prediction of antigenicity, allergenicity prediction, evaluation of toxicity profiles, and analysis of population coverage, as well as modelling of vaccine constructions prior to experimental validations.

Through utilizing multiple computational tools, the screening and optimization of epitope candidates that bind to major histocompatibility complex (MHC) molecules with high affinities were examined as *in silico* vaccine design for PLK1. Further, the immunogenic and physicochemical properties were also evaluated through publicly available databases and web servers such as the Immune Epitope Database (IEDB), VaxiJen, AllerTop, ToxinPred, and so on. Moreover, the multi-epitope-based vaccines (MEVs) were engineered by appropriate engagement of B-cell epitopes, cytotoxic T lymphocytes (CTL) and helper T lymphocytes (HTL) with suitable adjuvants and linkers to enhance the immunogenicity. Concurrently, the modelling of vaccine structures and subsequent analysis of molecular docking and structural dynamic behaviour with immune receptors such as toll-like receptors evaluate the stability of the designed vaccines. Furthermore, codon optimization, followed by *in silico* cloning, and the evaluation of the constructed vaccine’s immunomodulatory potential of the selected peptides determine the effective interactions with host systems.

Through acknowledging the clinical importance and oncogenic relevance of PLK1 in various types of cancers, efforts to employ an MEV that specifically targets PLK1 may enable an effective strategy in immunotherapy. Thus, the main objective of the current study is to utilize comprehensive immunoinformatic methods to design and evaluate MEVs against the PLK1 protein. Further, the availability of these open-sourced predictive software’s and algorithms could fasten the research on immunotherapeutic option against cancer, individualization, faster adaptation, and cost cutting in the field of clinical therapeutics.

## 2 Materials and methods

### 2.1 Computational resources

AllerTOP v2.1 (Allergenicity Prediction Tool) (lhttps://www.ddg-pharmfac.net/allertop_test/), BepiPred v2.0 (B-cell Epitope Prediction Tool) (https://tools.iedb.org/bcell/), C-ImmSim (Computational Immune System Simulator) (https://kraken.iac.rm.cnr.it/C-IMMSIM/index.php), ERRAT (Protein Structure Verification Tool for Non-Bonded Atomic Interactions) (https://saves.mbi.ucla.edu/), ExPASy ProtParam (Expert Protein Analysis System-Protein Parameters Tool) (https://web.expasy.org/protparam/), GROMACS (GROningen MAchine for Chemical Simulations) (https://manual.gromacs.org/current/download.html), HDOCK (Hybrid Protein–Protein Docking Algorithm) (http://hdock.phys.hust.edu.cn/), IEDB (Immune Epitope Database and Analysis Resource) (https://www.iedb.org/), JCat (Java Codon Adaptation Tool) (https://www.jcat.de/), NetMHCIIpan 4.1 EL (Neural Network–Based MHC Class II Binding Prediction Tool) (https://tools.iedb.org/mhcii/), NetMHCpan 4.1 EL (Neural Network–Based MHC Class I Binding Prediction Tool) (https://tools.iedb.org/mhci/), PDBsum (Protein Data Bank Structure Summary Database) (https://www.ebi.ac.uk/thornton-srv/databases/pdbsum/Generate.html), PROCHECK (Protein Structure Stereochemical Quality Check Tool) (https://saves.mbi.ucla.edu/), PSIPRED (Position-Specific Iterated Protein Secondary Structure Prediction) (https://bioinf.cs.ucl.ac.uk/psipred/), RCSB PDB (Research Collaboratory for Structural Bioinformatics Protein Data Bank) (https://www.rcsb.org/), SnapGene (Molecular Cloning and DNA Visualization Software), ToxinPred (Toxic Peptide Prediction Tool) (http://crdd.osdd.net/raghava/toxinpred/), tr-Rosetta (Transform-Restricted Rosetta Protein Structure Prediction Method) (https://yanglab.qd.sdu.edu.cn/trRosetta/), UniProtKB (Universal Protein Knowledgebase) (https://www.uniprot.org/help/uniprotkb), and VaxiJen v2.0 (Vaccine Antigenicity Prediction Server) (https://www.ddg-pharmfac.net/vaxijen/VaxiJen/VaxiJen.html).

### 2.2 Prediction of B-cell epitopes

**FIGURE 1:**
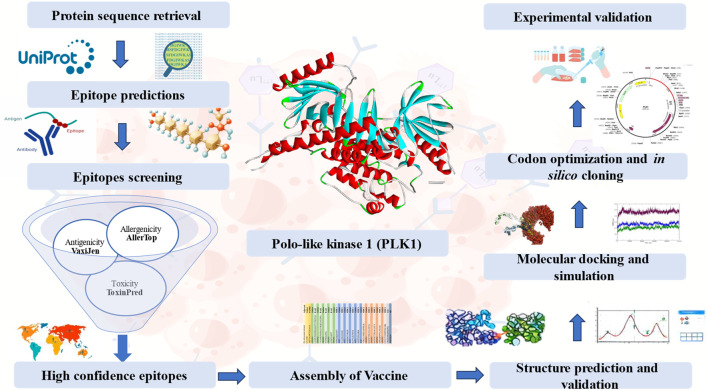
Schematic representation of overall workflow.

To design a vaccine targeting PLK1, it is necessary to predict antigenic determinants that are capable of eliciting cell-mediated and humoral immune responses. Primarily, the linear B-cell epitopes were identified using IEDB (Immune epitope database) resource (Vita et al., 2025). This public repository is utilized for predicting binding sites of antibodies that help in designing vaccines as well as being employed for other diagnostic and therapeutic applications. The amino acid sequence of PLK1 (UniProtKB ID: P53350) was subjected to the BepiPred linear epitope prediction 2.0 tool that is available in IEDB (Jespersen et al., 2017). The appropriate epitopes were selected through applying default parameters, high antigenicity score, sequence length, and surface accessibility area. Further, the overlapping and repeated sequences were removed to ensure non-redundant epitope profiling. The schematic representation of overall workflow is given in Figure 1.

### 2.3 Prediction of T-cell epitopes

To stimulate cellular immune responses, HTL (helper T lymphocytes) and CTL (cytotoxic T lymphocytes) epitopes were predicted using IEDB resource. Initially, the high binding affinity of CTL and HTL epitopes that adhere to MHC molecules (class I and class II) were screened through NetMHCpan 4.1 EL and NetMHCIIpan 4.1 EL predictors in the IEDB database (Reynisson et al., 2020). To ensure the broader immune activation potential, the peptides were selected using the following selection criteria. The selection criteria include the maximum amino acid length (above 5), low IC_50_ value (≤500 nM), and low percentile score (≤0.5). Subsequently, a more stringent filtering step was implemented to identify high-confidence epitopes, corresponding to an IC_50_ of less than 50 nM and a percentile score of 0.5% or lower. This approach ensured the selection of the most promising candidates for additional experimental validation (Subramani et al., 2026). Concurrently, the shorter sequences were excluded from the PLK1 vaccine constructions, as their immune modulation potential is poor. Thus, the peptides having low minimum inhibitory concentration, low percentile ranks, multi-allelic binding affinity, and high immunogenicity scores were utilized as high-affinity binders towards MHC class I/II molecules and selected for further immunogenicity evaluation.

### 2.4 Evaluation of epitopes antigenicity, allergenicity and toxicity

To evaluate the immunogenicity and safety profiles of the screened B- and T-cell epitopes, various comprehensive immunoinformatic tools were used. Primarily, the antigenic capacity of the B-cell and T-cell epitopes were predicted through VaxiJen v2.0 tool (Doytchinova and Flower, 2007). It is utilized for predicting protective antigens, tumor antigens, and subunit vaccines based on alignment-independent algorithms and followed with partial least square algorithms. By querying the peptide sequences, selecting the appropriate target (tumor), and selecting the default threshold value (0.5), the high-scored antigenic peptides were selected for PLK1 vaccine design. Following antigenicity, the epitopes were further screened for their allergenicity and toxicity profiles using AllerTOP v 2.1 and ToxinPred v 3.0 web sources (Dimitrov et al., 2014; Gupta et al., 2013). The AllerTOP tool utilizes K-nearest neighbour algorithms in order to classify the peptides into allergen and non-allergen. In contrast, the ToxinPred tool utilizes ensemble machine learning algorithms to classify the epitopes into toxic and non-toxic classes. The peptides that satisfying the immunological profiles such as non-allergenicity, antigenicity, and non-toxicity criteria were utilized for vaccine construction.

### 2.5 Prediction of population coverage

To ensure the selected vaccine candidates are able to induce protective immune responses across diverse groups, population coverage was performed through IEDB resource (Bui et al., 2006). Since human leukocyte antigens (HLA) are highly polymorphic and exhibit variability across diverse and ethnic groups, evaluating the distributed T-cell epitope-HLA binding profiles is essential in estimating effective immune responses. This tool utilizes a scoring-based approach to screen the set of epitopes based on human HLA-genotypic frequencies. For this process, the selected CTL and HTL epitopes were queried according to their MHC class (I/II) and HLA locus, such as HLA-A, HLA-B, HLA-DMA, HLA-DMB, and so on. From the analysis, the epitopes that covered higher global coverage and India populations were further prioritized for MEV construction.

### 2.6 Construction of primary MEVs and physiochemical characterizations

The selected B-cell epitopes and the high population covered CTL and HTL epitopes were assembled into MEV construction using suitable adjuvants and linkers. The adjuvant, namely, 50s ribosomal subunit protein L7/L12 (UniProt KB ID: P0A7K2) was added to induce both humoral and cellular immunity as well as to promote stronger activation of antigen-presenting cells (APCs). Further, the well-known linkers such as EAAAK, GPGPG, and AAY were added in between the epitopes to conjugate the vaccine structure as well as to improve antigen processing, maintain structural integrity, and enhance immunogenic efficiency. The linker EAAAK was utilized to create structural separation between adjuvant and epitopes as well as to prevent steric hindrances. In contrast, GPGPG and AAY linkers were utilized between helper T-cell to enhance the presentation of MHC class II molecules and CTL epitopes to facilitate the MHC class I presentation. Further, the TAT sequence (GRKKRRQRRRPQ) was incorporated to facilitate intracellular delivery of the vaccine construct. It enhances antigen processing via the MHC class I pathway, thereby promoting CTL-mediated immune responses against tumor cells (Zhou et al., 2025). Additionally, at the end of MEV construction, 6x His tag (HHHHHH) sequences were added to enable affinity purification. Then, the structurally determined MEV was examined for physiochemical and immunological characterization.

To examine the physicochemical characterizations of the MEV, the Expasy ProtParam, an online web resource was utilized (Gasteiger et al., 2005). Through this tool, molecular weight of the designed vaccine, amino acid composition profiles, theoretical isoelectric point (pI), extinction co-efficient, estimated half-life across different species, GRAVY metric, instability index, aliphatic index, and other parameters were calculated. Concurrently, the MEV was assessed for toxicity, antigenicity, and allergenicity profiles using VaxiJen, AllerTOP, and ToxinPred tools.

### 2.7 Structure prediction and validation

The constructed MEV’s secondary and tertiary structures were predicted through PSIPRED v 4.0 and trRosetta web servers (Du et al., 2021). PSIPRED utilized two-stage feed-forward neural network algorithms to predict high-accuracy secondary structure of the queried amino acid sequences (McGuffin et al., 2000). From the analysis, the locations of alpha helices, beta sheets, and coils were determined with high accuracy rates. In contrast, trRosetta utilized a deep residual-convolutional network algorithm along with energy minimization steps to model the 3D structures of the queried sequences. It works on identifying homologous sequences, detecting the evolutionary constraints, and then predicting geometric relationships such as distance between the amino acid residues as well as dihedral and planar angles. The 3D-generated vaccine was further evaluated for structural refinement to enhance the stereochemical quality.

For the model validation, PROCHECK, and ERRAT tools were utilized (RomanA et al., 1996; Laskowski et al., 2012; Colovos and Yeates, 1993). The PROCHECK works on the basis of calculating the backbone dihedral angles (phi and psi) of the queried 3D protein and identifying the favourable and unfavourable regions by plotting a Ramachandran plot. Subsequently, ERRAT analyzes the non-bonded atom-atom interactions in the queried 3D models of the proteins. From the analysis, the overall quality factor was determined. If the quality factor was found to be greater than 90, then the structure was considered a high-quality and reliable model. By using these structural validation tools, highly refined and reliable models were analyzed prior to docking analysis.

### 2.8 Molecular docking with immune receptors

To evaluate the molecular interactions between immune receptors such as toll-like receptors (TLR2 and TLR4) and designed PLK1 vaccine, binding energy was calculated using HDOCK tool. These receptors are playing a key role in enhancing antigen presentations as well as initiating innate immune responses in host systems. For the docking, the crystallographic 3D structures of the innate receptors such as TLR2 and TLR4 proteins were downloaded from the RCSB PDB database with IDs of 2Z7X (2.10 Å) and 2Z63 (2.00 Å) (Burley et al., 2023). The Protein-protein docking was performed utilizing the HDOCK (Yan et al., 2020). It uses a rigid-body docking program that uses integrated template-based modeling with fast Fourier transform sampling to determine the binding scores of the complexes and the potential docking poses of the targeted proteins. By querying the PDB structure files, HDOCK generated top-ranked docking models based on the binding affinity between the proteins. Further, the molecular interaction between the protein-protein docking were visualized through PDBSUM (Laskowski et al., 2018). It provides a pictorial representation of the interactions between the macromolecules, such as hydrogen bonds and non-bonded contacts. Through uploading the protein-protein complex files, the tool examined the stability and biological relevance of the proteins.

### 2.9 Analysis of structural dynamic behaviours of PLK1-TLR receptors

The structural stability and conformational dynamic behaviour were analyzed for constructed MEV and TLR complexes. The molecular dynamic simulation (MDS) was performed using GROMACS 2026. 0 software for the duration of 100 ns (Abraham et al., 2015). It is an open-source software that is utilized for analyzing the structural behaviour of biological macromolecules such as nucleic acids, ligands, and proteins. Molecular motions at an atomic level were calculated based on Newton’s equations of motion. For the MDS analysis, MEV and MEV-TLR complexes underwent parametrization using the CHARMM27 all-atom force field. Subsequently, the system was solvated using a water model such as TIP3P, and they were placed in a cubic simulation box. Then, the system was neutralized using appropriate counterions such as sodium/calcium ions. Prior to equilibration, energy minimization steps were carried out to remove the steric hindrances and unfavourable interactions. By employing the steepest descent algorithm, the system was neutralized until the maximum force was covered below 1000 kJ/mol/nm. Following that, NVT (constant number of particles, volume, and temperature) and NPT (constant number of particles, pressure, and temperature) ensembles were performed for 100 ps to equilibrate the system. In the NVT, constant temperature was maintained at 300 K using a thermostat, whereas in the NPT, the constant pressure was maintained at 1 bar using a barostat. After equilibrating the system, the final production of MDS was performed for 100 ns. From the MDS, the post-simulation trajectories were analyzed for calculating root mean square deviation (RMSD), radius of gyration (ROG), and solvent accessible surface area (SASA) to determine the structural stability of the MEV-immune receptor complexes.

### 2.10 Immune simulation profile of PLK1 vaccine

The immunogenic profile of the designed MEV was analyzed through C-ImmSim (Laubenbacher et al., 2024; Rapin et al., 2010). It is an online simulation tool, which is predominately utilized for evaluating the immunogenicity of the potential vaccine candidates. It utilized a machine learning approach along with Monte Carlo simulations to calculate the different immune events, such as antibody production, cell population dynamics, and cytokine levels. For the immune simulation, the default random seed (12,345), 1,000 simulation steps (approximately 333 days), and 10 simulation volumes in arbitrary computational units were applied for reproducibility and activation of immune systems. Further, the host HLA alleles were selected based on the high-binding affinity predictions. That includes, HLA-A*33:01, HLA-A*02:03, HLA-B*40:01, HLA-B*58:01, HLA-DRB1*01:01, and HLA-DRB1*07:01. Then, three injections in a time interval of 0th day, 28th day, and 58th day were added to evaluate the long-term immune responses. From the immune simulation, the output parameters, such as primary and secondary antibody responses, T-cell population, macrophage activity, dendritic cell activity, concentrations of cytokines and interleukins, and memory cell formations were analyzed to ensure the constructed vaccine’s potential application in cancer immunotherapy.

### 2.11 Codon optimization and *in silico* cloning of PLK1 vaccine

To analyze the heterologous expression of the constructed MEV, codon optimization and *in silico* cloning were performed using JCat and SnapGene tools (Grote et al., 2005; Dey et al., 2022; Sarker et al., 2019). JCat is utilized for optimizing the gene of interest in the desired organism through minimizing the rare codon usage, as they cause mRNA instability and premature termination. In contrast, SnapGene is a standalone software that is utilized for PCR-based cloning, restriction cloning, and annotating DNA sequences (plasmids, genomes, and vectors) as well as predicting DNA restriction fragments through agarose gel simulation. For the optimization, the FASTA sequence of MEV was subjected to the JCat, and a gene expression organism such as *Escherichia coli* K12 was selected. Following that, CAI (codon adaptation index) and GC (guanine and cytosine) content were calculated to estimate the translational efficiency of the constructed vaccine. Concurrently, the codon-optimized gene sequences were inserted into the SnapGene software for simulating recombinant plasmid construction. For the *in silico* cloning, the restriction enzyme sites such as NdeI and XhoI were inserted at the N- and C-terminals of the queried gene sequence, and the suitable pET-28 (+) vector was used for incorporating the optimized gene. Then, the final recombinant construct was analyzed for proper orientation, reading frame alignment, and absence of frame-shift mutations for subsequent expression and purification examinations.

### 2.12 *In vitro* evaluation

To evaluate the immunomodulatory potential of selected epitopes in PLK1, the SKBR3 cell line was utilized. As our previous study reported that the elevated levels of PLK1 expression were significantly observed in human breast adenocarcinoma cell lines, such as in SKBR3, compared to other cell lines (Munieswaran and Manickam, 2026). Thus, it serves with the experimental validation of PLK1-targeted immunotherapy. Initially, the SKBR3 cells were obtained from NCCS (National Centre for Cell Science), Pune, India, with the catalogue number of ATCC® HTB-30™. Then, the cells were cultured in 10% FBS (fetal bovine serum) supplemented Dulbecco’s modified Eagle medium (DMEM) and maintained at 37 °C in the humidified 5% CO_2_ incubator. Subsequently, the cells were treated with top-scored selected CTL epitopes in the concentration of 10 μg/mL for 48 h. Then, the total RNA was extracted from treated and untreated cells using RNAiso Plus reagent (Takara Bio Inc., Japan) according to the manufacturer’s protocol. Concurrently, cDNA was synthesized through ABScript II cDNA first-strand synthesis kit (Abclonal, China) through the manufacturer’s protocol. For the qRT-PCR (quantitative reverse transcriptase-polymerase chain reaction) analysis, Genious 2X SYBR Green Fast qPCR mix (Abclonal, China) was utilized according to the manufacturer’s instructions (Zhou et al., 2025; Vinodhini and Kavitha, 2025). The primers utilized for quantitative analysis were GAPDH (housekeeping control) (F: 5′-GGAGCGAGATCCCTCCAAAAT-3′ and R: 5′-GGCTGTTGTCATACTTCTCATGG-3′) and PLK1 (F: 5′-CACAGTGTCAATGCCTCCAA-3′ and R: 5′-TTGCTGACCCAGAAGATGG-3′). All these primers were synthesized through custom oligosynthesis with HPSF purification per base by Xetra Biosolution, India. All these *in vitro* experiments were conducted in triplicate as technical replicates to ensure analytical precision. Data were represented as mean ± standard deviation (SD). Statistical analysis between treated and untreated groups was performed through Student’s two-tailed t-test in Origin 2026 software. The *p*-value < 0.0001 was considered as statistically significant.

## 3 Results

### 3.1 Retrieval of potential B cell epitopes

**FIGURE 2:**
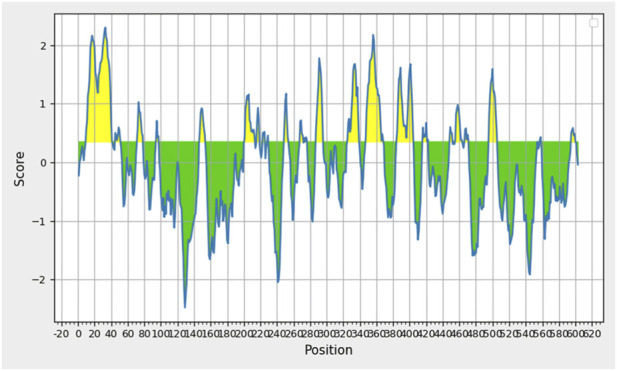
Linear B-cell epitope prediction chart. Yellow color represents epitope regions and green color represents non-epitope regions.

For the construction of the PLK1 vaccine, initially the linear B cell epitopes were predicted using the BepiPred tool. Table 1 illustrates the prediction profile of B cell epitopes. The Figure 2 illustrates the amino acid positions in the PLK1 sequence and their epitope prediction score. The graph represented the non-epitope regions (green-colored peaks) and linear B-cell epitopes (yellow-colored peaks) based on threshold values. By analyzing the results, a total of 25 potential linear B-cell epitopes were scored above the threshold value, such as 0.5. The list of predicted B-cell epitopes given in Supplementary Table S1. To further ensure the structural stability and biological relevance of suitable candidates for MEV construction, the B-cell epitopes consist of minimum seven amino acid residues were considered for antigenicity, allergenicity, and toxicity analysis. Through analyzing the immunological properties, four epitopes exhibited the highest antigenicity score, and which also did not exhibit any allergenicity and toxicity profiles. These shortlisted B-cell epitopes were utilized for PLK1 vaccine construction.

**TABLE 1:** Selected B-cell epitopes through various tools.

Start	End	Peptide	Length	AllerTop	VaxiJen	VaxiJen score	ToxinPred
287	295	QTDPTARPT	9	Non-Allergen	Antigen	0.71	Non-toxin
347	368	LENPLPERPREKEEPVVRETGE	22	Non-Allergen	Antigen	0.57	Non-toxin
384	404	VNASKPSERGLVRQEEAEDPA	21	Non-Allergen	Antigen	0.93	Non-toxin
496	505	NITPREGDEL	10	Non-Allergen	Antigen	0.56	Non-toxin

### 3.2 Retrieval of potential CTL and HTL epitopes

Following B-cell prediction, T-cell epitopes that bind to MHC molecules were screened using the tools available in the IEDB. By applying the selection criteria, 253 CTL epitopes were identified as potential MHC class I binding peptides. In contrast, around 698 potential MHC class II binding peptides were identified. The list of CTL and HTL epitopes are given in Supplementary Tables S2, S3. These epitopes were predicted based on the binding affinity (IC_50_) to various HLA alleles and percentile scores. Subsequently, the epitopes were scrutinized for immunological potential using various bioinformatic tools and web resources. From the initially screened dataset, a stringent filtering step was applied using low percentile scores (≤0.5%) and low IC_50_ values (<50 nM) to identify high-confidence epitopes, resulting in 152 CTL and 260 HTL epitope candidates. The subsequent safety profiling, which focused on non-allergenicity and non-toxicity, narrowed down the epitope candidates to 21 CTL and 27 HTL epitopes. From this selection, ten strong antigenic binding epitopes targeting MHC class I (CTL) and MHC class II (HTL) molecules were chosen for vaccine construction. The list of strong binders selected for vaccine construction along with its immunological properties are given in Tables 2, 3.

**TABLE 2:** Selected cytotoxic T lymphocytes epitopes through various tools.

HLA types	Start	End	Length	Peptide	IC_50_ (nM)	AllerTop	VaxiJen	VaxiJen score	ToxinPred
HLA-A*02:03, HLA-B*40:01	375	384	10	SDMLQQLHSV	20.17	Non-allergen	Antigen	1.56	Non-toxin
HLA-A*30:01	575	584	10	ELASRLRYAR	18.75	Non-allergen	Antigen	1.06	Non-toxin
HLA-A*11:01, HLA-B*58:01	48	57	10	RSRRRYVRGR	21.42	Non-allergen	Antigen	0.95	Non-toxin
HLA-A*02:03, HLA-A*33:01	507	516	10	RLPYLRTWFR	5.03	Non-allergen	Antigen	0.94	Non-toxin
HLA-A*03:01, HLA-A*30:01	466	475	10	SSHPNSLMKK	28.34	Non-allergen	Antigen	0.94	Non-toxin

*denotes the HLA allele subtype associated with the predicted epitope.

**TABLE 3:** Selected helper T lymphocytes epitopes through various tools.

HLA types	Start	End	Length	Peptide	IC_50_ (nM)	AllerTop	VaxiJen	VaxiJen score	Toxin pred
DRB1*01:01, 04:01, 04:05, 07:01, 09:01, DRB3*02:02, DRB5*01:01	587	601	15	VDKLLSSRSASNRLK	23.31	Non-allergen	Antigen	0.63	Non-toxin
DRB1*01:01, 15:01, 09:01, DPA1*01:03, 03:01, 02:01	557	571	15	RDFRTYRLSLLEEYG	34.85	Non-allergen	Antigen	0.65	Non-toxin
DRB1*01:01, 15:01, 09:01, 07:01, DPA1*01:03, 03:01, 02:01	556	570	15	KRDFRTYRLSLLEEY	31.34	Non-allergen	Antigen	0.64	Non-toxin
DRB1*01:01, 13:02, 09:01, 07:01	269	283	15	SIPKHINPVAASLIQ	30.8	Non-allergen	Antigen	0.61	Non-toxin
DRB1*01:01, 13:02, 09:01, 07:01, 04:05	460	474	15	ESYLTVSSHPNSLMK	32.4	Non-allergen	Antigen	1.08	Non-toxin

*denotes the HLA allele subtype associated with the predicted epitope.

### 3.3 Population coverage analysis of selected T cell epitopes

**FIGURE 3:**
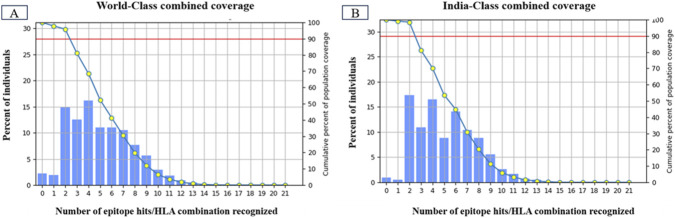
Number of epitopes hit analyzed through population coverage. **(A)** World-class combined coverage and **(B)** India-class combined coverage.

Utilizing IEDB’s population coverage prediction tool, the population coverage was performed to determine the global and regional applicability of the screened T-cell epitopes. This tool estimates the proportion of each individual in the given population based on the HLA allele distribution frequencies. By querying the combined class coverage of selected MHC class I and MHC class II binding epitopes, 97.82% of the world population was covered, highlighting their ability to exhibit immune responses among the majority of the population. The predicted average epitope hit (5.13) as well as the percentile coverage (2.4) demonstrated that at least 90% of the population were expected to recognize a minimum of two to three epitope-HLA combinations. Similarly, the region-specific population coverage showcased that the combined T-cell epitopes covered 99.1% of the population in India. Further, the average number of epitope hits and percentile scores were observed to be 5.22 and 2.49, suggesting a maximum number of individuals may have the capability to recognize multiple epitope-HLA combinations from the given set. The population coverage and number of epitope hits are given in Table 4 and the diagrammatic representation is given in Figure 3. Overall, the population analysis covered a large proportion of individuals, suggesting the selected T-cell epitopes may exhibit strong immunogenic potential and wide applicability across various ethnic groups, which have been utilized for multi-epitope vaccine (MEV) construction.

**TABLE 4:** Population coverage analysis using selected T-cell epitopes.

Population area	Coverage	Average hits	Minimum number of epitope hits (pc90)
World	97.82%	5.13	2.4
India	99.1%	5.22	2.49

pc, percentile coverage.

### 3.4 Construction and characterization of the MEV

**FIGURE 4:**
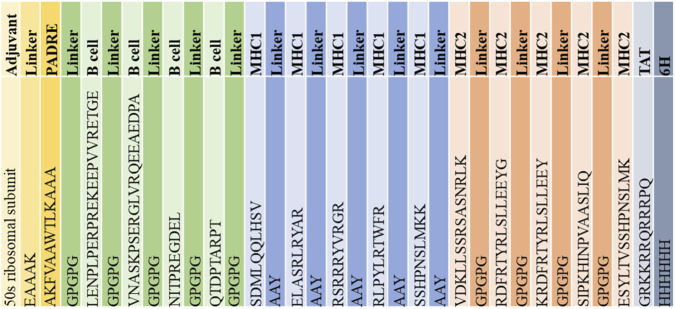
Structure of constructed PLK1 vaccine using selected epitopes and linkers.

The screened B-cell and T-cell epitopes were assembled in the MEV construction using appropriate linkers and adapters in order to maintain structural integrity and subsequent antigen presentation. Figure 4 illustrates the assembling of MEV using selected T and B cell epitopes. Table 5 showcases the physiochemical and immunological properties of constructed PLK1 vaccine. The final designed MEV consists of 404 amino acids, and the predicted molecular weight of PLK1 vaccine and theoretical isoelectric point were observed to be 43.25 KDa and 9.57. The total number of positively charged (Asp + Glu) and negatively charged (Arg + Lys) residues were 49 and 61, respectively. The extinction coefficient was computed to be 30,370 M^-1^ cm^-1^, suggesting the expression and purification of the protein could be implemented fast. Conversely, the estimated half-life of the MEV was observed to be 30 h in mammalian reticulocytes *(in vitro*), >20 h in the yeast expression system, and >10 h in *E. coli*. The calculated instability index and aliphatic index were found to be 9.88 and 73.07 respectively, which indicates the constructed PLK1 vaccine is stable, and its thermostability behaviour is considerably good. Further, the predicted GRAVY value was −0.576, which categorizes the vaccine as hydrophilic and subsequently enhances the interactions with the host immune system. Further, the predicted immunological profile of constructed MEV as an antigen, was classified as a non-allergen and non-toxin. Comprehensively, the evaluation of various physicochemical and immunologic properties exhibited that the designed MEV was categorized as stable and had strong antigenic potential. Eventually, making it suitable for structural modeling and further vaccine development analysis.

**TABLE 5:** Physicochemical and immunological properties of constructed PLK1 vaccine.

Properties	Predictions
Number of amino acids	404
Theoretical isoelectric point (pI)	9.57
Molecular weight	43,252.06 KDa
Total number of negatively charged residues (Asp + Glu)	49
Total number of positively charged residues (Arg + Lys)	61
Formula	C_1898_H_3057_N_571_O_572_S_7_
Total number of atoms	6,105
Extinction coefficient	30,370 M^-1^ cm^-1^
Estimated half-life	30 h (mammalian reticulocytes, *in vitro*)
	>20 h (yeast, *in vivo*)
	>10 h (*Escherichia coli*, *in vivo*
Instability index	39.88 (stable)
Aliphatic index	73.07
Grand average of hydropathicity (GRAVY)	−0.576
Antigenicity	Antigen (0.6138)
Allergenicity	Non-allergen
Toxicity	Non-toxic

### 3.5 Structural modelling and validation of PLK1 vaccine

**FIGURE 5:**
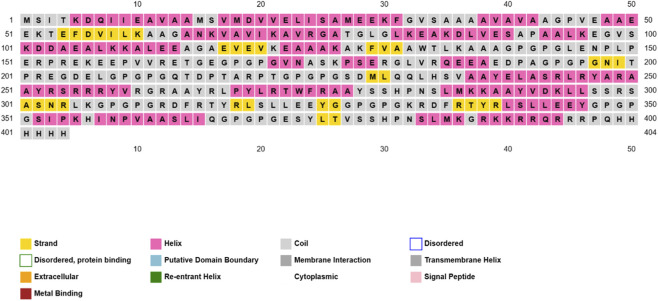
Secondary structure prediction of constructed PLK1 vaccine through PSIPRED tool.

**FIGURE 6:**
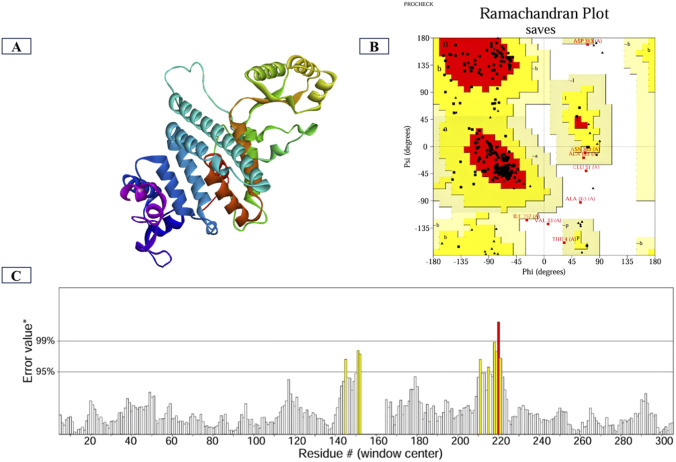
Tertiary structure prediction and validation of constructed PLK1 vaccine. **(A)** 3D visualization of PLK1 vaccine, **(B)** Ramachandran plot and **(C)** ERRAT profile.

The prediction of the secondary structure of the MEV revealed that the presence of multiple helical segments, beta-strands, and coil regions, confirming its good structural stability upon having low flexibility linkers and loop regions. Thus, the design further enhances the folding and organization of PLK1 vaccine’s structure. The secondary structure predicted through PSIPRED is illustrated in Figure 5. By knowing the structural elements, the PLK1’s vaccine 3D structure was built through homology modelling. The well-refined model from trRosetta was selected and subsequently analyzed for its structural validation. The stereochemical quality of the vaccine determined through plotting the Ramachandran graph revealed that 89.3% of residues were abundant in the most favoured regions, whereas the remaining residues were located in the additional allowed regions (8.3% residue), generously allowed regions (0.9% residues), and disallowed regions (nearly 1.5% residues). These results demonstrated favourable conformations as indicated by the localization of the majority of the backbone dihedral angles (phi and psi). Concurrently, the structure validation by the ERRAT determined that the overall quality factor was observed to be 96.875, which was well above threshold value (greater than 50%), suggesting that the designed vaccine had good structural quality, better resolution with favourable atomic interactions. The 3D visuvalization and structural validation are given in Figure 6. The analysis through structural modelling and validation indicated that the constructed PLK1 vaccine exhibited greater stereochemical quality and structural reliability. Thus, making it suitability for further downstream analysis with immune receptors.

### 3.6 Molecular interactions between immune receptors and PLK1 vaccine

**FIGURE 7:**
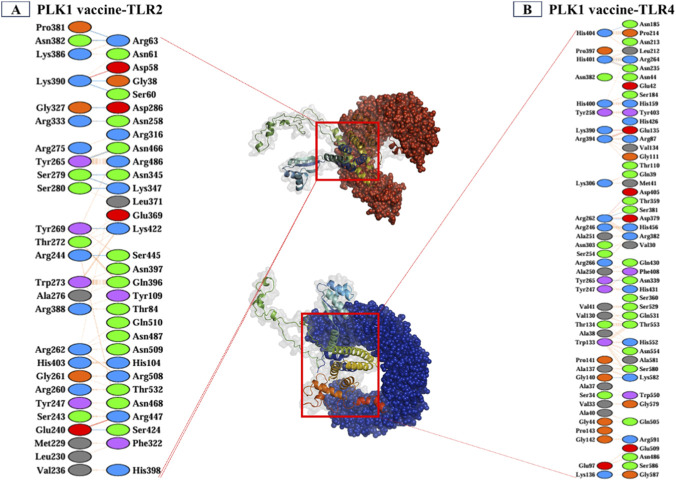
Interaction profile between constructed PLK1 vaccine and immune receptors. **(A)** 2D interactions between PLK1 vaccine and TLR2 receptor and **(B)** 2D interactions between PLK1 vaccine and TLR4 receptor.

The molecular-level interactions between toll-like receptors (TLR2 and TLR4) and PLK1 vaccine were analyzed through HDOCK. The top-ranked docking complexes with the lowest binding energy models were considered for further analysis. The predicted binding energies between the constructed PLK1 vaccine and immune receptors were found to be −353.83 kcal/mol (for PLK1 vaccine-TLR2 receptor) and −402.83 kcal/mol (for PLK1 vaccine-TLR4 receptor). Conversely, the confidence score for both complexes was observed to be greater than 0.98, suggesting the stable interactions. Further, the visualization of the docking structures demonstrated that the PLK1 vaccine-TLR4 complex (13 H-bonds) exhibited a greater number of hydrogen bond interactions compared to the PLK1 vaccine-TLR2 complex (7 H-bonds). The presence of high hydrogen bond interactions between the PLK1 vaccine and the TLR4 complex showcased strong intermolecular stabilization, which may lead to the activation of a Th1-mediated immune response. Further, non-bonded interactions and salt bridges between the PLK1 vaccine and TLR2 was found to be 391 and 4 respectively. Whereas, the PLK1 vaccine-TLR4 complex displayed a total of 260 non-bonded interactions and 2 salt bridge interactions. The analysis of residue level interactions revealed that the residues such as ASN382, LYS390, TYR265, ARG262, and TYR247 in the PLK1 vaccine commonly interacted with TLR receptors. The 2D interaction profile and interface residues between constructed PLK1 vaccine and immune receptors are given in Figure 7. Here, the presence of shared interface residues suggested that the constructed PLK1 vaccine may be able to interact with multiple immune receptors, thereby enhancing immune activation.

### 3.7 Molecular dynamic simulation of vaccine-TLR complexes

**FIGURE 8:**
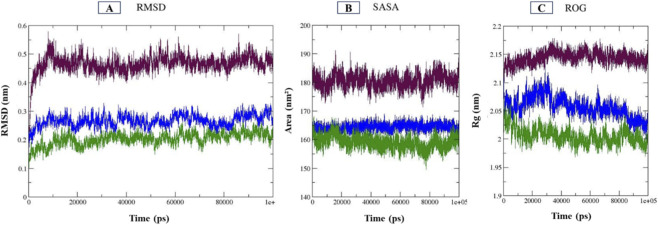
Structural dynamic behavior analysis between constructed PLK1 vaccine and immune receptors. **(A)** RMSD-root mean square deviation, **(B)** SASA-solvent accessible surface area, **(C)** ROG-radius of gyration, magenta color-PLK1 vaccine, blue color-PLK1 vaccine-TLR2 receptor and green color-PLK1 vaccine-TLR4 receptor.

To evaluate atomic-level interactions and structural stability between the designed PLK1 vaccine and TLR complexes, the molecular dynamic simulations (MDS) was carried out for 100 ns using GROMACS. Figure 8 illustrates the structural dynamic analysis between constructed PLK1 vaccine and immune receptors. By analyzing the stability using the RMSD graph, it was revealed that both complexes and the PLK1 vaccine alone reached an equilibration state after the initial stage of simulations. The graph displayed that the PLK1 vaccine (magenta) exhibited lower fluctuations ranging from 0.42 nm to 0.56 nm. The PLK1 vaccine-TLR2 complex (blue) maintained stable RMSD in the range between 0.24 and 0.31 nm, while the PLK1 vaccine-TLR4 complex (green colour) maintained slightly lower deviation in the range of 0.15–0.23 nm. These findings demonstrated that the PLK1 vaccine maintained stable conformations with immune receptors throughout the 100 ns simulation period.

The analysis of solvent accessibility surface area displayed that the PLK1 vaccine alone had exposures to surrounding solvent within the area of 170–190 nm^2^. While the PLK1 vaccine-TLR2 and PLK1 vaccine-TLR4 complexes maintained minimum to stable fluctuations after 20 ns in the range between 150 and 168 nm^2^. The lower SASA indicates that more compact, tightly packed and buried interface residues between the PLK1 vaccine and TLR receptors.

Furthermore, the analysis of the compactness of the protein structure using ROG plots demonstrated that the PLK1 vaccine-TLR2 and PLK1 vaccine-TLR4 complexes maintained low ROG values. Precisely, the complexes-maintained stability after 20 ns simulations, and the observed ROG was 2.02–2.1 nm (for PLK1 vaccine-TLR2 and 1.97–2.03 nm (PLK1 vaccine-TLR4). In contrast, the PLK1 vaccine provided ROG values in the range of 2.11 nm–2.17 nm. These analyses revealed that the PLK1 vaccine contained a slightly expanded and partially unfolded structure, while the interactions with immune receptors contained tightly packed and folded structures.

Overall, the MDS profile revealed that the PLK1 vaccine with innate immune receptors maintained structurally stable conformations under physiological conditions when compared to the constructed PLK1 vaccine. Conversely, the interactions with TLR4 protein remain stable, indicating a favourable predicted binding pose that may enhances potential receptor recognition, which could be associated with Th1-mediated immune responses.

### 3.8 Immune simulation of the PLK1 vaccine

**FIGURE 9:**
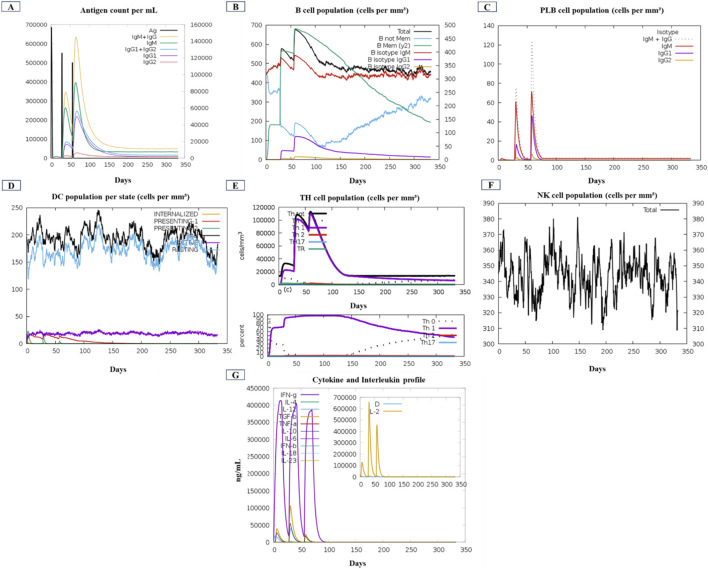
Immune profile of constructed PLK1 vaccine. **(A)** Immunoglobulin production, **(B)** B-cell population, **(C)** Plasma B-cell population, **(D)** Dendritic cell population, **(E)** T-helper cell population, **(F)** Natural killer cell population, and **(G)** Cytokine profile.

The designed PLK1 vaccine’s immunogenic potential was evaluated through the C-ImmSim. The overall immune simulation of the constructed PLK1 vaccine is shown in Figure 9. After administration of the vaccine, the concentration of antigen rapidly decreases due to clearance and prominent increase in primary and secondary immune responses. Precisely, the primary and secondary immune responses were dominated with IgM antibodies (380,000 counts/mL) and followed by IgG antibodies (Figure 9A). Whereas the combined IgM and IgG antibodies elevated at the concentration of approximately 630,000 counts/mL. This event reflecting enhanced antigen neutralization and long-term immune response. Further, the number of B-cell populations was increased after booster injections, which accounted for approximately 650–700 cells per mm^3^ (Figure 9B). Further, the observation of increased memory B cells suggested long-term humoral immunity. Concurrently, the formation of plasma B cells suggests effective recognition and neutralization of antigen as indicated by activation of elevated IgM (∼70 cells per mm^3^) and IgG (∼8–45 cells per mm^3^) antibody productions (Figure 9C).

Further, the antigen processing and presenting them to T cells were accessed through analysis of dendritic cell populations (Figure 9D). The graph demonstrated that the total number of dendritic cells fluctuated in the range of 160–240 cells per mm^3^. Among these, resting dendritic cells occupied a major proportion of the population, whereas the antigen-presenting dendritic cells peaked during the initial booster doses (∼30–50 days). This event suggested that the constructed PLK1 vaccine facilitates an effective immune response as indicated by antigen uptake, processing, and presentation. In contrast, the natural killer cell populations were observed in the range between 310 and 380 cells per mm^3^, indicating surveillance of the innate immune system remained stable, while activating the adaptive immune system (Figure 9F).

Additionally, the elevated levels of T helper cells were observed during immune simulation. Precisely, the total of T helper cell populations peaked in the range of 10,000–11,000 cells per mm^3^ and the majority of the population was occupied by Th1 cells (around 10,000 cells per mm^3^) (Figure 9E). Thus, it is evident that the constructed MEV may predominately activate a Th1-mediated immune response. Subsequently, the interferon (IFN)-gamma productions were observed in elevated levels, followed by tumor necrosis factor (TNF)-alpha, interleukin (IL)-4, and IL-12. IFN-gamma production was observed in the range between 350,000 and 400,000 ng/mL, suggesting further efficiency of immune activation and proliferation (Figure 9G).

Collectively, these findings predicted that the designed PLK1 vaccine may able to activate both humoral and cell-mediated immune responses as indicated by enhanced antigen presentation, recognition, and elevated cytokine profiles.

### 3.9 *In silico* cloning of the PLK1 vaccine

**FIGURE 10:**
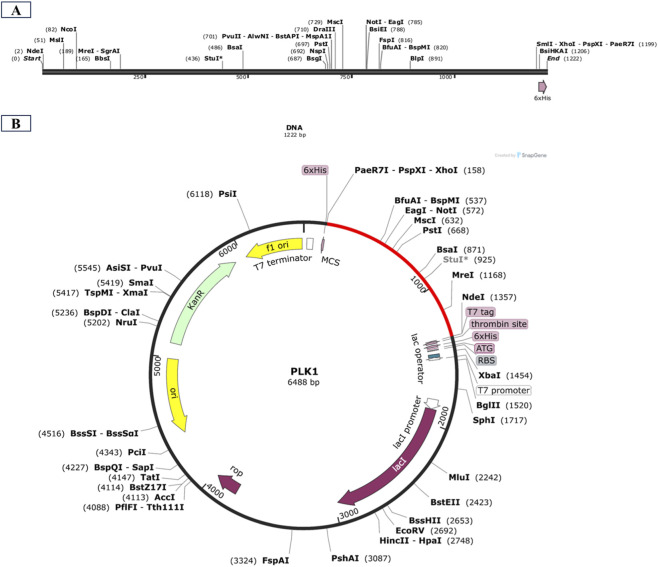
Codon optimization and *in silico* cloning of constructed PLK1 vaccine. **(A)** Restriction sites of designed vaccine, and **(B)** *In silico* cloned vaccine in pET28a (+) vector.

To ensure the expression of the designed PLK1 vaccine was efficient, codon optimization was evaluated using the *E. coli* K12 bacterial system with the help of the JCat tool. From the figure, the optimized gene sequence was observed to be 1,222 bp in length, while the CAI (codon adaptation index) value and GC content were observed to be 0.88% and 73%. The optimal range of the GC contents and high compatibility with the host system suggested that efficient gene expression was examined. Following that, the complete gene sequence was cloned using the pET expression vector by incorporating restriction enzymes at N- and C-terminals. Figure 10 illustrates the codon optimized and *in silico* cloned PLK1 vaccine in pET vector. After introducing the optimized gene in the appropriate cloning site, the gene expression was monitored and controlled by the T7 promoter. The final recombinant plasmid length was observed to be 6,488 bps. Thus, the integration of the optimized gene without disrupting the origin of replication and other essential vector elements suggested that the constructed MEV was suitable for high-level recombinant protein expression in the given *E. coli* system. Further, the 6xHis tag in the constructed MEV might further facilitate downstream analysis, especially purification of the recombinant protein expressions.

### 3.10 *In vitro* validation of PLK1-targeted CTL epitopes

**FIGURE 11:**
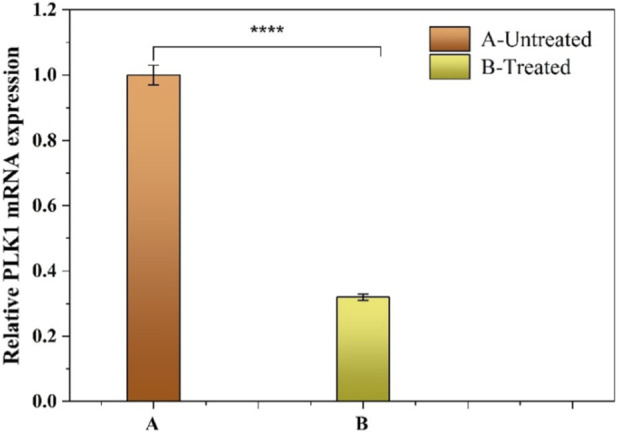
Relative PLK1 mRNA expression in SKBR3 cells following CTL epitope exposure. Data are represented as mean ± SD of technical triplicates. Group A represents the untreated control, while Group B represents the epitope-treated group. Significant statistical difference between the control and treated groups is indicated by ****p < 0.0001.

To assess the potential biological effects of the predicted CTL epitopes, preliminary *in vitro* validation was performed in SKBR3 cancer cell lines. Table 6 displays the qPCR analysis of PLK1 gene expression in SKBR3 Cells. The expression level of PLK1 was analyzed in treated and untreated SKBR3 cells using the ΔΔCt method. The untreated control group exhibited a mean ΔCt of 12.18 ± 0.025, while the epitope-treated group demonstrated a significant increase in ΔCt, with a mean value of 13.82 ± 0.017. Thus, resulting ΔΔCt of 1.64 indicates a clear reduction in the PLK1 mRNA levels. The relative fold change (2^−ΔΔCT^) was observed to be 0.32, which corresponds to a log2 fold change of −1.64, suggesting a 3.1-fold decrease in PLK1 expression in treated cells exposed to the CTL epitopes compared to untreated groups. The statistical analysis confirmed the significantly downregulated PLK1 expression (p-value <0.0001). The graphical representation of CTL treated and untreated groups are represented in Figure 11. The detailed raw Ct values are given in Supplementary Table S4. These findings are consistent with *in silico* predictions; however, further experimental investigations are required to evaluate their biological and immunological relevance.

**TABLE 6:** qPCR analysis of PLK1 gene expression in SKBR3 Cells.

Parameter	Value
ΔΔCt ( $\Delta Ct_{treated}-\Delta Ct_{control}$ )	1.64
Fold change ( $2^{-\Delta \Delta Ct}$ )	0.32
Log2 fold change ( $Log_{2}FC=-\Delta \Delta Ct$ )	−1.64
*p*-value	<0.0001
Fold decrease (1/fold change)	∼3.1
Regulation	Downregulated

ΔCt, Delta cycle threshold.

## 4 Discussion

Polo-like kinase 1 plays a central role in mitotic regulation and other essential cellular developmental processes. Dysregulation of PLK1 is frequently observed in numerous types of malignancies and becomes a poor prognosis, thus making it a suitable candidate for cancer and immunological therapy. The study reported that the overexpression of PLK1 protein affects overall survival and progression-free survival of the lung cancer patients. Concurrently, the elevated oncogenic signaling pathways were activated along with PLK1 in non-small cell lung cancer patients, thus leading to significantly lower survival probabilities (*p* = 0.0063) (Li et al., 2026). Our previous study reported that the overexpression of PLK1 observed in breast, liver, lung, kidney, and pancreatic cancer patients was indicated for the poor survival outcomes (Munieswaran and Manickam, 2025). Recent investigations on PLK1 downregulation showed that targeting PLK1 could potentially improve the immunotherapy and may perturb the tumor immune microenvironment (Kong et al., 2024). Precisely, upon targeting PLK1 has been reported to be associated with changes in antigen presentation and may influences adaptive immune responses. The experimental study conducted using nanoparticle-based immunotherapy targeting PLK1 in lung cancer improved the expression of MHC class I molecules, thereby recognizing CTL (Reda et al., 2022). Thus, it is identified that PLK1 is an important oncogenic target, and the antigen derived from it may contribute to improved antitumor responses in certain contexts.

To achieve the study’s objective, initially B and T cell epitopes with high antigenic properties were predicted. The computational analysis predicted a total of 14 epitopes for constructing vaccine which includes four B-cell epitopes, five CTL epitopes, and five HTL epitopes. These epitopes were characterized as non-toxic, non-allergenic and highly antigenic. The analysis of B cell epitopes plays a fundamental role in antibody-mediated immune response, which is essential for antigen neutralization and long-term immunity (Townsend et al., 2022; Braun et al., 2025). Conversely, the investigations on CTL epitopes are highly associated with recognizing tumor-associated antigens, thereby eliminating cancer cells. Similarly, the investigations on HTL epitopes play a crucial role in coordinating activated B-cell and CTL and cytokine secretions, thereby enhancing cell-mediated immune response (Kamali et al., 2025). Further, the analysis about population coverage revealed the applicability of the selected vaccine candidates. The selected T-cell epitopes covered global populations about 97.82%, suggesting that the epitopes were able to stimulate immune response across diverse ethnic groups. The investigations on HLA polymorphism through examining the population coverage significantly influence the antigen presentation and recognition of immune responses. The recent study reported that the T-cell epitope conserved in cancer vaccines covered global population above 90%, indicating the increased likelihood of vaccine-induced immunity (Zhou et al., 2025; Firuzpour et al., 2025). Additionally, the regional population was covered by 99.1%, thus confirming the regional applicability of the selected epitopes.

Utilizing the selected epitopes, the multi-epitope vaccine (MEV) was constructed using appropriate adapters and linkers, and subsequently its physicochemical and immunological properties were determined. The predicted properties indicated that the designed PLK1 was categorized as stable and hydrophilic, which may favour further downstream requirements. A previous study reported that the computationally designed HER2-NTGP96 vaccine is often categorized as hydrophilic, suggesting the vaccine has better interaction with water solvents (Atapour et al., 2020). They also reported that the instability of the designed vaccine was found to be greater than 40, which classifies the chimeric protein as slightly unstable, whereas the aliphatic index classifies it as stable under various temperatures. In contrast, the study generated a structurally stable PLK1 vaccine where the instability index was found to be below 40, suggesting the protein was stable and suitable for immunological analysis. Likewise, secondary structures predicted elevated levels of coils and followed by alpha helixes, indicating the presence of more abundant flexible regions that facilitate antigen accessibility by aligning proper protein folding. Further, 3D structural modelling and validation through Ramchandran plots revealed that most of the residues were occupied in the favoured regions. In addition, the high score of the ERRAT value also determined that the stereochemical quality of the PLK1 vaccine was considered as good and thus utilized for docking with innate immune receptors.

The construction of a vaccine targeting triple-negative breast cancer proteins showcased that the designed vaccines, such as beta-defensin-adjuvanted MEV, exhibited high binding affinity towards TLR4 receptors compared to TLR2 receptors (Zhou et al., 2025). The TLR4 receptors consist of extracellular leucine-rich repeat domains (ligand recognition), transmembrane domains, and intracellular toll domains that are responsible for activating pro-inflammatory cytokines and subsequent innate and adaptive immunity. In this context, the constructed PLK1 vaccine exhibited a favourable docking score (−402.83 kcal/mol) with the TLR4 receptor, along with multiple hydrogen bond interactions (approximately 13), indicating a stable predicted interaction that may be associated with TLR4 recognition and Th1-mediated immune responses. Moreover, the MDS demonstrated that compared to the candidate vaccine alone, the vaccine-bound innate immune receptors showed stable and advantageous conformational behaviour as analyzed through low RMSD, high compactness, and low solvent exposures (Subramani et al., 2026). This further supports the binding affinity analysis and together may be efficient for immune activation. The immunogenic potential was analyzed through performing immune simulation, which revealed the elevated level of IgM and IgG antibodies, increased number of B cell populations, and high amount of cytokine, especially INF-gamma productions. In comparison with previous findings, it was reported that the increased levels of interferon-gamma and interleukin-2 attained through the constructed vaccine may modulate adaptive and innate immune responses in the host body. The previous literature also reported that the designed cancer antigens produce strong INF-gamma and IL-2 secretions, and thus are associated with effective tumor antigen recognition and subsequent elimination by activating appropriate immune responses (Elshafei et al., 2024).

*In silico* cloning further confirmed the feasibility of the protein expression in the bacterial expression system (Roy et al., 2024; Wang et al., 2023). The codon-optimized genes exhibited significantly favourable codon adaptation index (CAI) and GC contents and were subsequently inserted into the pET expression vector. The results determined that the codon-optimized recombinant plasmid expression could be in elevated levels, and might enhance the efficient protein yield in the experimental validations. The analysis of the immunomodulatory potential of transcription factors such as myeloid zin finger 1, Mucin1, SRY-box transcription factor 9, keratin (Braun et al., 2025; Munieswaran and Manickam, 2025) and Twist-related protein one in triple-negative breast cancer progression revealed the significantly downregulated expression observed after exposure of CTL epitopes. The results indicated that a 1.8-to-2.4-fold decrease of targeted protein expression was observed in the treated groups (Zhou et al., 2025). In comparison with the current findings, the PLK1 gene expression decreased 3.1-fold after exposure of selected CTL epitopes in SKBR3 cells. This cancer cell line was selected based on the high proliferative activity resulting from PLK1, thus making them suitable for experimental model for evaluating regulatory effects and subsequent cellular responses (Munieswaran and Manickam, 2026). This experimental validation was restricted to SKBR3 cells as proof-of-concept model. However, further *in vitro* validations, including peripheral blood mononuclear cell (PBMC)-based assays, dendritic cell maturation, antigen presentation studies, and tumor immune co-culture models are required to evaluate the immunological relevance of the proposed PLK1 vaccine. In addition to that, functional assays such as cytokine profiling (IFN-gamma and IL-2 secretion), flow cytometric analysis of T-cell activation markers, and cytotoxicity assays will further enable a comprehensive evaluation of the vaccine’s suitability to be able to induce antigen-specific cellular and humoral immune responses. Furthermore, *in vivo* studies in appropriate animal models will be essential to validate the findings of the current investigations.

In spite of the promising findings from *in silico* and preliminary *in vitro* studies, several limitations should be considered for effective treatments. The observed downregulation of PLK1 in SKBR3 cells may suggest potential antigen modulation, a mechanism recognized as crucial for tumor immune evasion. Furthermore, the cancer vaccine design utilizing an immunoinformatics approach broadly relies on predictive algorithms and is not directly involved in antigen processing and tumor heterogeneity. Recent studies suggested that the development of successful vaccines largely depends on not only the selection of antigens but also effective delivery systems as well as effective immune activation strategies. For instance, the study utilized a lipid-based nanoparticle platform to effectively deliver the vaccine, thereby enhancing the antigen stability and immune responses (Zhang et al., 2022; Zhang et al., 2026). Besides that, targeting the tumor-associated proteins may express variability across different tumor types but may have the potential to modulate immune-related pathways, which could enable more targeted and effective modulation of immune responses in cancer therapies (Du et al., 2026). Therefore, to address the critical limitations, further studies incorporating immune-relevant models, evaluation of antigen stability in different tumor models, and the exploration of advanced delivery strategies are required to improve the translational potential of the proposed vaccine construct.

## 5 Conclusion

Overall, the current study identified PLK1 as a crucial cancer antigen, and a subsequent multi-epitope vaccine that targets PLK1 was constructed using immunoinformatic approaches. By arranging B cell and T cell epitopes with highly antigenic, non-allergenic, and non-toxic profiles, favourable physicochemical and immunological properties were exhibited. The structure modelling and validation further confirmed the stability of the designed PLK1 vaccine, while molecular docking and molecular dynamic simulation results suggested that the PLK1 vaccine-TLR4 complex exhibited favourable and stable interactions compared to the TLR2 receptor. Immune simulation also predicted that the designed PLK1 vaccine increases the production of elevated levels of antibodies, immune cells, and cytokines. The codon-optimized recombinant protein expression in the *E. coli* system showed efficient expression. These investigations indicates that the PLK1 vaccine may serve as a promising candidate for cancer immunotherapy. Further, experimental validation through appropriate animal models may prove the constructed PLK1 vaccine is able to activate potential immune signaling pathways, such as the activation of T cells and the enhancement of antibody responses, which are crucial for effective cancer immunotherapy.

## Data Availability

The datasets presented in this study can be found in online repositories. The names of the repository/repositories and accession number(s) can be found in the article/Supplementary Material.
